# Membrane-Based Microwave-Mediated Electrochemical Immunoassay for the In Vitro, Highly Sensitive Detection of Osteoporosis-Related Biomarkers

**DOI:** 10.3390/s18092933

**Published:** 2018-09-04

**Authors:** Hye Youn Kim, Shinobu Sato, Shigeori Takenaka, Min-Ho Lee

**Affiliations:** 1 School of Integrative Engineering, Chung-Ang University, 84 Heukseok-Ro, Dongjak-Gu, Seoul 06974, Korea; hykimbio@cau.ac.kr; 2 Department of Applied Chemistry, Research Center for Biomicrosensing Technology, Kyushu Institute of Technology, Fukuoka 804-8550, Japan; shinobu@che.kyutech.ac.jp (S.S.); shige@che.kyutech.ac.jp (S.T.)

**Keywords:** membrane-based microwave-mediated electrochemical immunoassay (MMeEIA), bone turnover marker, osteoporosis, C-terminal cross-linked telopeptide of type I collagen (CTx), osteocalcin (OC), parathyroid hormone (PTH), N-terminal propeptide of type I collagen (P1NP)

## Abstract

Highly sensitive and multiplexed in vitro detection of osteoporosis-related biochemical markers were carried out based on the membrane-based microwave-mediated electrochemical immunoassay (MMeEIA), where we can dramatically reduce the sample preparation time by shortening the incubation time of conjugation to obtain sensitive detection based on three dimensional conjugation of antibodies with target antigens in nylon membrane disk. C-terminal cross-linked telopeptide of type I collagen (CTx), Osteocalcin (OC), parathyroid hormone (PTH), and N-terminal propeptide of type I collagen (P1NP), which can be utilized to monitor the progress of osteoporosis, were quantified using their corresponding antibody immobilized in membranes. Coefficient of variations in this intra- and inter-assays were within 8.0% for all markers. When compared with data obtained from clinically used standard equipment (Roche modular E170), their coefficients of determination, *R*^2^ values, are mostly more than 0.9. They show that the results obtained from MMeEIA are in good agreement with that from the conventional clinical instruments.

## 1. Introduction

Osteoporosis is well-known as a disease causing the continuous loss of bone mineral density (BMD) which eventually leads to the deterioration of bones and increased risk of bone fracture. Globally over 200 million women have been affected and approximately 8.9 million fractures have been reported due to this disease [1]. Currently the diagnosis of osteoporosis is highly dependent on the X-ray imaging of 2-D structure of bones, for example, bone densitometry or dual-energy X-ray absorptiometry (DEXA), which can be further interpreted as density of bone minerals [2]. However, the progress of osteoporosis and frequent monitoring of efficacy of treatment using medicine is still dependent of changes in levels of the related bone turnover markers of bone formation and resorption which are tightly linked in BMD conditions [3,4,5,6,7]. In addition, immunoassays for bone turnover markers provide complementary management of dynamic changes of BMD due to its easiness to access, convenient, and no risk of radiation. Among the number of related biomarkers, Osteocalcin (OC) and the N-terminal propeptide of procollagen type I (P1NP) have been used as a bone formation biochemical marker and the C-terminal cross-linked telopeptide of type I collagen (CTx) has been commonly used as bone resorption marker. The level of serum osteoporosis was measured to be relatively higher in post-menopausal woman with osteoporosis than the normal [8,9]. Due to its high affinity to calcium, the increased level of osteocalcin present in serum is attributable to the deficiency of calcium and phosphorous on which the bone formation process is highly dependent [10]. P1NP, the product of osteoblast, was also widely recommended as a bone formation marker because of its stability in serum and less variability between serum samples [11,12]. The level of P1NP in serum is highly related with the rate of the bone formation because it is released before it is incorporated into the extracellular matrix. The fragment from C-telopeptide of type I collagen, CTx, has been studied for monitoring the resorption rate during osteoclasts. In contrary to the aforementioned bone formation markers, CTx is an indicator of the rate of degradation of the synthesized collagen. Therefore, the quantification of each of the bone formation and resorption markers has been used as indirect indicators of bone related diseases including osteoporosis. Other than those well-known markers, Parathyroid Hormone (PTH) is known as the regulator of serum calcium and phosphate metabolism in bone and can act as an osteoporosis-related biomarker in parathyroid disorders. In this regard, we believe that membrane-based electrochemical detection technology is a promising candidate for the simple method of multiplexed immunoassays. Electrochemical immunoassays have been studied in many ways to increase sensitivity, cost effectiveness, efficiencies in pretreatment, amplification, and simple handling [13,14,15,16]. Recently, the electrochemical immunoassay of osteoporosis has been studied regarding the sensing electrode carrying gold nanoparticles [17] or MoS_2_-graphene composite [18]. In this study, we developed a simple electrochemical immunoassay based on the microwave-mediated immobilization of antibodies and the antibody–antigen reaction. The use of a membrane for pretreatment and a custom-made electrode for measurement provide low cost, ease of use, and rapid preparation for the immunoassay of target proteins because the role of the membrane is for the pretreatment or formation of the antigen–antibody complex and that of the electrode is used only for measurement. Therefore, the same electrode can be used multiple times, which provides an advantage over the most commonly used electrodes that require target antibodies to be immobilized on it. A target conjugated electrode cannot be used more than once. In addition, reduced preparation time from immobilization of capture antibodies to last step of measurements gives better expandable applications than the use of precoated cartridges in that the kinds of target needed to be measured at the field can be easily applied.

The objectives of present work are: (i) to prepare a membrane disk (MD) in which target antibodies are placed; (ii) to fabricate custom-made electrodes to perform electrochemical assays via differential pulsed voltammetry (DPV); and (iii) to perform a multiplexed detection of such antigens and evaluate the method through comparison with results obtained from clinical instrumentation.

## 2. Materials and Methods

### 2.1. Reagents and Materials

Hydrophilic nylon membrane filters were purchased from Merck Millipore (Burlington, MA, USA). Among the candidate membranes, such as polyvinyllidene fluoride membrane [19], nitrocelluose [20], and MF membrane [21], the Nylone membrane was chosen because it showed good durability and stability in the membrane holder during the experiments. *p*-Aminophenyl phosphate (pAPP) was synthesized as described previously [22]. Monoclonal antibody to cross-linked CTx, biotin-linked polyclonal antibody to CTx, bovine serum albumin (BSA)-conjugated CTx, monoclonal antibody to P1NP, biotin-linked polyclonal antibody to P1VP, and ovalbumin (OVA)-conjugated P1NP were all sourced from Cloud-Clone Corp (Katy, TX, USA). OC monoclonal antibody, human OC full length protein, and anti-PTH antibody (biotin) were all sourced from Abcam (Cambridge, UK). OC Polyclonal Antibody, Biotin Conjugated was purchased from Bioss antibodies (Woburn, MA, USA). Mouse monoclonal PTH antibody and purified recombinant human PTH protein were purchased from Fitzgerald (North Acton, MA, USA). The neutravidin alkaline phosphate conjugated product (NA-ALP) was purchased from Thermo Scientific (Waltham, MA, USA). DNA LoBind deep-well plate was purchased from Eppendorf (Hamburg, Germany). All other chemicals were purchased from Sigma-Aldrich (St. Louis, MO, USA) or Life Technologies (Carlsbad, CA, USA). All reagents were of analytical grade, and used without further purification. The deionized water (DI water, >18 MΩ cm) used to prepare all solutions in this study was obtained from a water purification system. Antigens and antibodies used in this study is shown Table 1. 

### 2.2. Preparation of Membrane Disks

**Figure 1:**
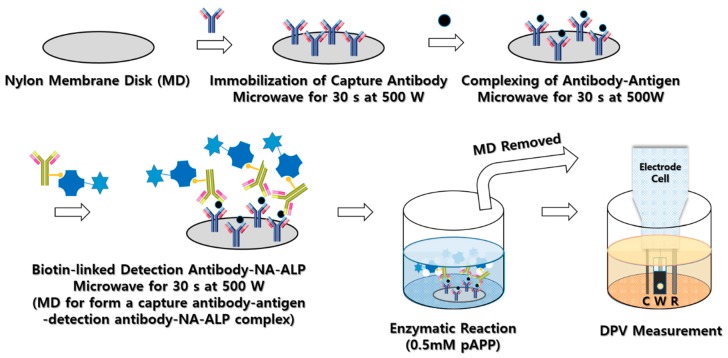
Schematic diagram showing the process of MMeEIA and surface modification of membrane disk for antibody binding.

Antibody was immobilized based on the protocol which have been modified for this study [21]. For the immobilization of capture antibodies, nylon-membrane filter disks (diameter: 6 mm) were prepared. Capture antibodies of 10 μg/mL were directly dropped on them followed by irradiation of microwave for 30 s at 500 watts. This immobilization processes significantly reduced the time of incubation which normally needs overnight incubation. Each membrane disk (MD) was then placed in a 1.5 mL centrifuge tube containing solutions of 0.5% casein in 1 × PBS (137 mmol/L NaCl, 8.1 mmol/L Na_2_HPO_4_, 2.68 mmol/L KCl, 1.47 mmol/L KH_2_PO_4_, pH 7.4) for blocking and then the tube was microwaved for 30 s at 500 watts. After incubation for 10 min, the disk membrane was removed from the tube and the capture antibody conjugated MDs were ready for the simultaneous pretreatment prior to electrochemical measurement. The capture antibody conjugated MD was placed on a custom-made holder tip (Figure S2) and then the holder was moved to the first low of the wells where MDs were immersed into the buffer solution (100 mM ammonium acetate, 0.05% Tween-20) and incubated for 20 min. 10 μL of separate target antigens of different concentrations or clinical samples were dropped on the MDs followed by microwave (30 s, 500 W) irradiation for fast antigen–antibody reaction. Then, the holder was moved to the third row of wells that contained 15 μL of 2.5 μg/mL target detection antibodies and 1.0 μg/mL NA-ALP in 100 μg/mL BSA, 10 mM HEPES (pH 7.4), 10 mM NaCl, 0.05% Tween-20 for the formation of a capture antibody–antigen-detection antibody-NA-ALP complexes and underwent microwave irradiation (30 s, 500 W). When the MD holders were moved to the fourth and fifth row of wells, in sequence, they underwent three times of up and down washes with 1 × PBS and 0.05% Tween-20 for 5 min. The membrane disks were then incubated for 30 min. The last row of 7 × 6 well titer had a solution of 10 mM KCl, 10 mM MgCl_2_, and 0.5 mM pAPP where enzymatic reaction process produces active *p*-aminophenol for further differential pulsed voltammetry (DPV) based quantification of antigens. The processes of membrane disk conjugation were depicted in Figure 1. The set ups for DPV measurements were −0.4 to 0.3 V for each initial and final potentials with scan rate of 50 mV/s and pulse amplitude of 0.05 V with pulse period of 0.05 s with increment of 0.005 V. The electrochemical cell included an Au electrode, an Ag/AgCl electrode (saturated 3 M KCl), and a platinum (Pt) electrode as the working, reference, and counter electrodes, respectively.

### 2.3. Custom-Made Well Titer

For the simultaneous multiple target measurements, we have also built a customized well type titer (7 × 6) as shown in Figure S1. Each column of the titer has 6 wells, which were composed of wells for buffer solution, the target analyte, or sample containing well (changeable), secondary Ab well, 1st washing well, 2nd washing well, and the last well for DPV measurements. Therefore, simultaneous pretreatments can be performed separately and separate electrode cells can be placed; the measurement of different target analytes using separate workstations (4 targets in this study) is also possible. In addition, we have a newly designed membrane disk holder in which the capture antibody conjugated membrane can be placed between the holder and its lid (Figure S2). In this experiment, we have used four holders for separate measurements.

### 2.4. Custom-Made Gold Electrode

Electrochemical immunoassays were performed with four separate CHI 660E potentiostats/Galvanostats (CH Instruments, Inc., Austin, TX, USA) with a custom-made Au-deposited glass chip (Figure S3).

The manufacturing of the customized Au-chip employed the conventional MEMS process which consists of: (1) metal deposition (Cr/Au = 200 Å/2000 Å); (2) photoresist patterning; (3) metal etching; (4) PR pattering for insulation and curing; and finally (5) dicing for chip preparation. Overall the size of the Au-chip is 20 mm × 4 mm (Length × Width) with a 2 mm diameter circular working electrode.

### 2.5. In Vitro Measurement

We measured serum CTx, OC, P1NP, and PTH using the MMeEIA. All measurements were performed on serum samples that were collected beforehand and stored at −70 °C. Thirty osteoporotic patients were identified from Seoul National University Bundang Hospital in the age range of 50 to 60 years and were postmenopause. All subjects were free of hormone therapy and medicine which may hinder the measurements. Serum samples from these patients were used to investigate the concentration of CTx, OC, P1NP, and PTH level using the MMeEIA and compared with a standard electrochemical luminescence immunoassay (ECLIA) device from MODULAR ANALYTICS, the E170 (Roche Diagnostics, GmbH, Mannheim, Germany).

## 3. Results

### 3.1. DPV Measurement

**Figure 2:**
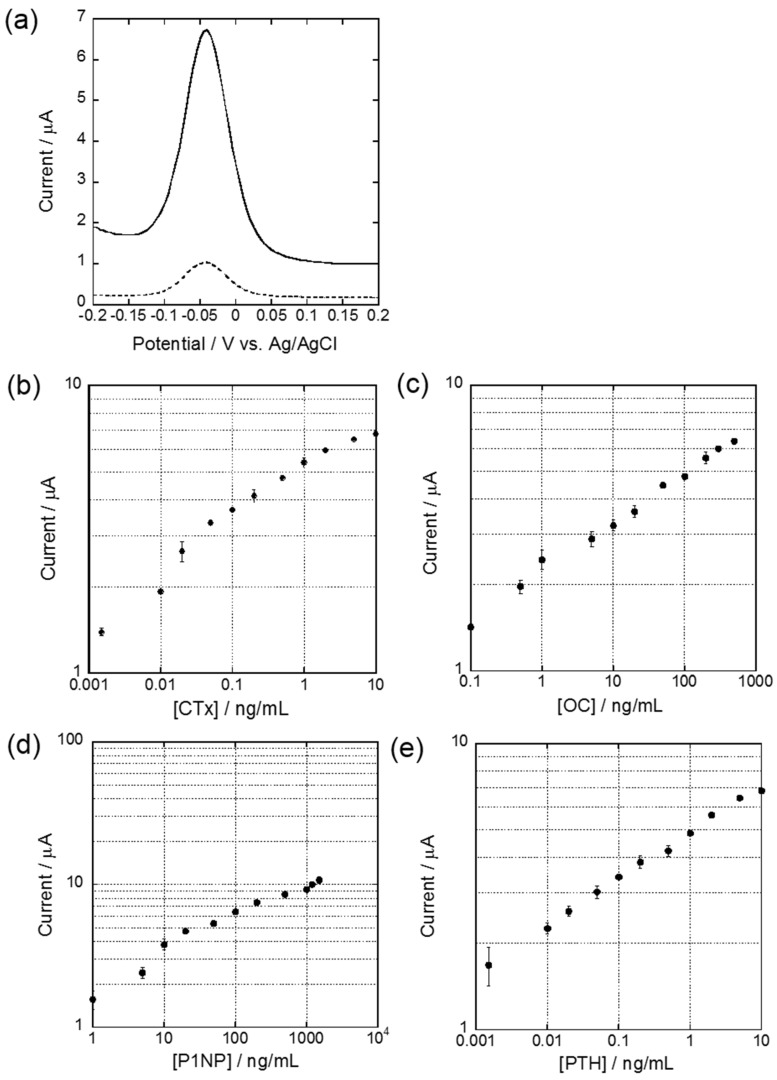
(**a**) Differential pulse voltammograms of a single analyte CTx (Solid line: 10 ng/mL, Dotted line: negative control). Standardization curve between the peak current and the concentration of (**b**) CTx, (**c**) OC, (**d**) P1NP, and (**e**) PTH by MMeEIA, respectively. All experiments were carried out three times. The bar graph represents the mean ± SD (*n* = 3).

We used a nylon membrane filter (0.45 μm pore size) as MD which has a good protein capacity (150 μg/mL) and durability so that it can be easily placed into the custom-made membrane holder. According to a previous report [21], the capture antibody was immobilized on MD with 30 s microwave irradiation at 500 watts followed by 10-min incubation at room temperature. In addition, the total duration from sample pretreatment to the detection of osteoporosis-related biomarkers was less than 90 min. Therefore, MMeEIA could significantly reduce the time of preparation when compared with the conventional ELISA-based analytical method. After completing preparations, MDs were removed and Au-chips were instead placed into the last row of wells for the final measurements. For the DPV measurements, we used the pAPP substrate and alkaline phosphatase (ALP) labeled antibodies to generate the electrically active product *p*-aminophenol (pAP) in an amount which we could quantify the level of target antigens. Figure 2a shows MMeEIA in the case of 10 ng/mL CTx concentration; a typical example of DPV results. As a first step, Au-chips fabricated using MEMS procedure on same or different wafer were evaluated to examine their reproducibility after pretreatment with different concentrations of CTx, OC, P1NP, or PTH. Then, the intra-assay and inter-assay were then performed using those fabricated chips. Evaluation of intra-assay (five times per test) variation with concentrations was calculated to be CVs 1.25–5.69%, 2.63–7.67%, 0.51–7.01%, and 1.02–8.16% for CTx, OC, P1NP, and PTH, respectively and tabulated in Table 2. CVs for the inter-assay variation using five different MDs and Au-chips were analyzed to be 1.87–7.85%, 1.20–6.59%, 0.29–4.94%, and 1.20–3.45% for their respective markers. Both intra-assay and inter-assay variations for all markers were approximately less than 8.0%.

The standard concentrations for calibration selected for this study are 0.001–10 ng/mL, 0.1–500 ng/mL, 0.1–1200 ng/mL, and 5.0–5000 ng/mL for CTx, OC, P1NP, and PTH, respectively [23,24,25]. All selected bone turnover markers were pretreated using the membrane disks simultaneously and each immunoassay result was performed using Au-chips. In addition, measurements of each marker were evaluated for further clinical use. As can be seen in Figure 2b–e, the measurements of all markers (CTx, OC, P1NP, and PTH) represent good linearity against concentrations and their working ranges were indicated as 0.01–6 ng/mL, 0.5–300 ng/mL, 5.0–1200 ng/mL, and 0.005–5 ng/mL for CTx, OC, P1NP, and PTH, respectively.

The lower limit of detection (LLOD) was calculated to be 1 pg/mL, 100 pg/mL, 1 ng/mL, and 1 pg/mL for CTx, OC, P1NP, and PTH, respectively, according to the capable performance of our method. The higher limit of quantification (HLOQ) was measured to be 10 ng/mL, 500 ng/mL, 1500 ng/mL, and 10 ng/mL based on the highest measurement with standard deviation less than 20% in Table 2.

### 3.2. Cross Reactivity

**Figure 3:**
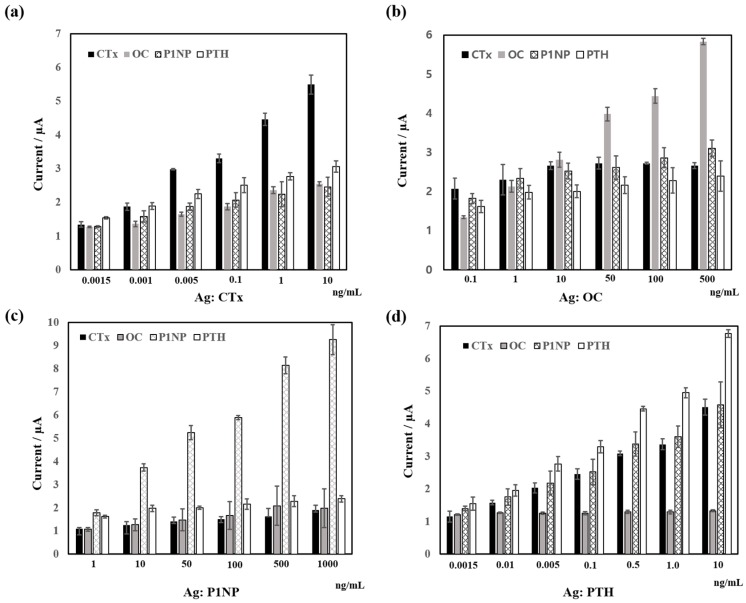
Results of cross-reactivity: (**a**) CTx antigen (0.01–10 ng/mL range) interaction with OC, P1NP, and PTH pair antibodies; (**b**) OC antigen (0.1–1000 ng/mL range) interaction with CTx, P1NP, and PTH pair antibodies; (**c**) P1NP antigen (1–1000 ng/mL range) interaction with CTx, OC, and PTH pair antibodies; and (**d**) PTH antigen (0.01–10 ng/mL range) interaction with CTx, OC, and P1NP pair antibodies.

Figure 3 shows for each marker the standard plot of the cross-reactivity between target proteins and other turnover markers. As can be seen in Figure 3, target antigen–antibody bindings showed relatively low-affinity binding to other markers resulting in lower current signals. The measurements schemes are target antibody coated MDs were incubated with other nontarget markers. For example, in the case of OC antigens against other markers, we have prepared antibody coated MDs and their corresponding pair labeled antibodies such as CTx, P1NP, and PTH in this case; the complex was incubated with OC antigens. For the measurements of changes in signal, Au-chips were employed and DPV was performed. Figure 3 shows that cross reactivity of antigens with other related nonpairing antibodies. More specifically, Figure 3 shows, (a) CTx antigen (0.01–10 ng/mL range) interaction with OC, P1NP, and PTH antibodies; (b) OC antigen (0.1–1000 ng/mL range) interaction with CTx, P1NP, and PTH antibodies; (c) P1NP antigen (1–1000 ng/mL range) interaction with CTx, OC, and PTH antibodies; and (d) PTH antigen (0.01–10 ng/mL range) interaction with OC, CTx, and P1NP antibodies. The results show that the measured interfering signals were relatively low in the range above 1 ng/mL. The cross-reactivity rate is also calculated and shown in Table 3. We have set up the antibody pairs and measured the electrical signal changes of target antigens. Results showed that the paired antibodies are well matched with their target antigens except in the case of PTH antibodies against CTx and P1NP.

### 3.3. Analysis of Biomedical Markers in Human Serum

**Figure 4:**
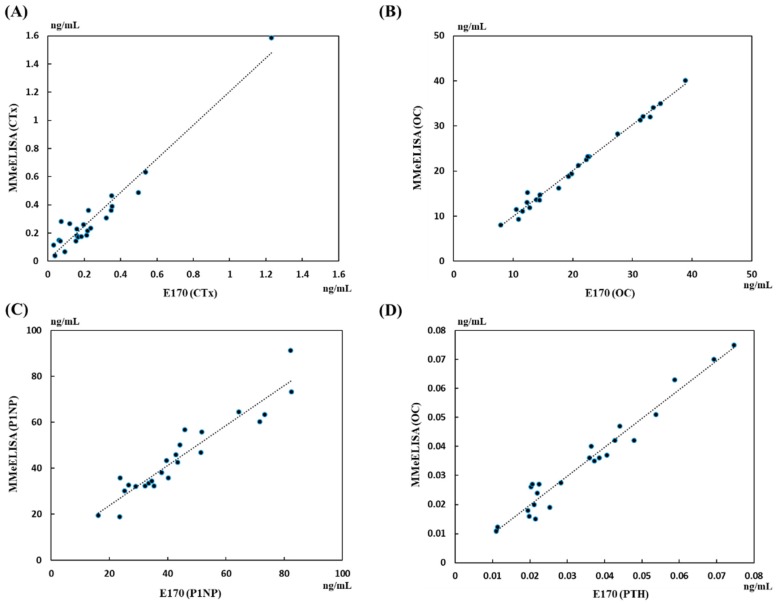
Measurement results of clinical samples using MMeEIA method and their correlation with the standard results using E170 (Roche): (**A**) CTx; (**B**) OC; (**C**) P1NP; (**D**) PTH. The Pearson correlation factor between measured concentration and standard results were 0.94, 0.96, 0.88, and 0.98, respectively.

Bone turnover markers from patient sera were also evaluated using the E170 in Figure 4. The evaluated marker concentrations using E170 were used for reference standards. The measured marker concentrations using Au-chips showed high correlation with those E170; correlation coefficients of *R*^2^ > 0.90 (*P* < 0.01). The correlation factor of P1NP is relatively lower (*R*^2^ = 0.88) than other results because we have used fabricated recombinant fragmented antigen and corresponding antibodies for the measurement. The native antigen present in serum can bind with a limited number of epitopes of the fabricated antibodies used in this study. The other markers of CTx, OC, and PTH showed good correlation as 0.94, 0.96, and 0.98, respectively. This demonstrates that the developed MMeEIA is in good agreement with the standard measurement device E170, indicating that the system can be effective in measuring the levels of antigens in human serum samples.

As can be seen in Table 4, all serum samples used in these tests were collected from patients with either osteoporosis or osteopenia. All subjects’ T scores were obtained from eight spines using the DEXA system. Most of the CTx concentrations were less than 0.6 ng/mL, which can be expected in postmenopausal women with osteopenia/osteoporosis [24]. In addition, the concentration ranges of P1NP and PTH displayed in this study were 16.22–82.5 ng/mL and 10.9–74.6 pg/mL, respectively. These measured results from two biomarkers in this study showed good agreements with previous results [24]. Lowered concentrations of OC measured in this study (7.9–38.9 ng/mL) also matched well with the previous results [8]. Therefore, our newly developed system is able to provide an easy, fast, and precise supporting tool for monitoring the progress of osteoporosis.

## 4. Conclusions

We have developed a simple, fast, cost-effective, and sensitive platform to measure bone turnover markers which is highly useful in determining not only for the progress of osteoporosis, but also in monitoring the efficacy of medicine in treatment of osteoporosis. For this purpose, we have chosen widely-used bone turnover markers such as CTx, OC, P1NP, and PTH and built a titer kit and mesh type membrane holder platform (Figure S1) for simultaneous pretreatment and electrochemical-based measurement. For the signal generation, we have employed ALP conjugated antibody and pAPP for the quantification of target proteins using the differential pulse voltammetry (DPV) method. We have obtained the standard calibration curve having inter- and intra-assays of 8% and showing good linearity in the concentrations of each corresponding markers with measurable LLOD and HLOQ. In addition, comparison with clinically used equipment (E170, Roche) showed good correlation coefficients; the correlation factor of P1NP is relatively lower (*R*^2^ = 0.88) than other results because we have used fabricated recombinant antigen, antibodies. However, both measured and reference CTx, OC, and PTH had Pearson correlation coefficients of 0.94, 0.96, and 0.98, respectively. This result implies that the developed MMeEIA is in good agreement with the result from standard measurement using a E170 device, and also indicates that the system can be effective in measuring the levels of antigens in human serum samples. Overall, the specific ranges of concentration measured in this study further indicate that the changes of biochemical markers of serum from patients with osteoporosis/osteopenia could be used as a monitoring method for the progress of osteoporosis and that our newly developed system could also be utilized as a supportive tool. The subject of our related future study will be the development of a fully equipped automated instrument using the previously described sensor and cartridges that can quantify the concentrations of the aforementioned turnover markers automatically in serum. In addition, small size circuit boards for multiple DPV should be installed to minimize the overall platform (figure is not included).

## Figures and Tables

**Table 1:** Capture antibodies, antigen, and detection antibodies.

	Capture Antibodies	Antigen	Detection Antibodies
CTx	Monoclonal antibody to cross-linked CTx	BSA conjugated CTx	Biotin-linked polyclonal antibody to CTx
OC	OC monoclonal antibody	Human OC full length protein	OC polyclonal antibody, biotin conjugated
P1NP	Monoclonal antibody to P1NP	OVA conjugated P1NP	Biotin-linked polyclonal antibody to P1NP
PTH	Mouse monoclonal PTH antibody	Recombinant human PTH protein	Anti-PTH antibody (Biotin)

**Table 2:** Analytical evaluation of MMeEIA for bone turnover markers.

	Intra-Assay CV (%)	Inter-Assay CV (%)	LLOD	HLOQ
CTx	1.25–5.69	1.87–7.85	1 pg/mL	10 ng/mL
OC	2.63–7.67	1.20–6.59	100 pg/mL	500 ng/mL
P1NP	0.51–7.01	0.29–4.94	1 ng/mL	1500 ng/mL
PTH	1.02–8.16	1.20–3.45	1 pg/mL	10 ng/mL

**Table 3:** Comparison of cross-reactivity rate between biochemical markers.

Cross Reactivity Rate ^1^ (%)					
	Target Antigen	CTx	OC	P1NP	PTH
Pair Antibody					
CTx		100	30.8	28.4	36.6
OC		13.1	100	28.5	17.1
P1NP		10.7	12.2	100	19.0
PTH		64.4	2.3	60.9	100

^1^ Cross-reactivity rate: (Tmax 2 − Tmin 3)/(Cmax 4 − Cmin 5) × 100, where: Tmax 2: Current value at maximum target concentration; Tmin 3: Current value at minimum target concentration; Cmax 4: Maximum current at positive control; Cmin 5: Minimum current at negative control.

**Table 4:** Measured T score of spines of patients (1st to 8th, *n* = 24).

No.	1st	2nd	3rd	4th	5th	6th	7th	8th
1	2.0	2.1	2.1	2.6	**−0.3**	**−0.6**	1.1	0.9
2	−2.8	**−2.2**	**−1.4**	−0.7	**−1.9**	**−2.2**	−0.7	**−1.2**
3	−0.9	−0.3	−0.6	**−1.2**	**−1.2**	**−1.6**	−0.3	−1.2
4	**−1.3**	**−1.2**	−0.7	−0.7	−0.6	**−1.5**	0.1	0.1
5	**−1.9**	−2.6	−3.0	−2.5	**−1.4**	**−1.6**	−0.3	−0.8
6	−0.6	−0.4	−−0.1	−0.8	−1.0	−1.2	0.4	0.2
7	**−1.3**	**−1.4**	−0.8	0.0	**−1.2**	**−1.6**	0.0	−0.1
8	−2.7	−3.7	−3.2	−2.8	**−1.1**	**−1.7**	−1.2	−0.8
9	−1.0	−0.1	−0.1	−0.1	**−1.4**	**−2.2**	−0.1	−0.5
10	−1.0	**−1.1**	**−1.2**	**−1.1**	−0.9	**−1.7**	−0.9	−0.7
11	**−2.2**	**−2.4**	−2.6	**−1.1**	**−2.0**	−2.5	**−1.0**	**−1.4**
12	−0.6	0.0	−0.5	0.1	−1.0	−1.4	0.3	0.2
13	**−1.2**	**−1.5**	**−1.4**	−1.0	**−1.7**	**−2.4**	−0.8	−1.0
14	**−1.6**	**−1.2**	**−1.7**	−0.8	−0.6	**−1.6**	0.0	0.1
15	**−1.6**	0.0	0.5	**−1.6**	**−1.6**	**−2.1**	−0.9	**−1.1**
16	**−1.5**	−0.8	−0.3	−1.0	−0.5	−2.5	**−1.1**	**−1.1**
17	**−1.4**	−2.9	−2.8	**−1.7**	−0.5	**−1.7**	−0.2	−0.3
18	−0.8	−0.7	0.5	0.7	−1.9	−2.7	0.4	−0.8
19	**−1.6**	**−1.7**	**−1.6**	**−1.5**	**−1.3**	**−1.7**	**−1.1**	−0.9
20	−2.8	**−2.2**	**−1.7**	**−2.1**	**−1.5**	−2.5	−0.9	**−1.2**
21	−3.2	−2.7	−3.0	−2.7	**−1.7**	**−1.9**	**−1.3**	**−1.4**
22	**−2.3**	−2.7	−2.9	**−1.3**	**−2.1**	**−2.4**	**−1.4**	**−1.3**
23	**−2.3**	−3.0	−3.0	−2.7	**−1.3**	**−2.1**	**−1.3**	**−1.1**
24	**−1.3**	**−2.4**	**−2.0**	**−2.2**	**−1.9**	−2.5	−0.6	−0.5

Normal T ≥ −1.0, Osteopenia −2.5 < T < −1 (bold), Osteoporosis T ≤ −2.5 (highlighted).

## Supplementary Materials

The following are available online at http://www.mdpi.com/1424-8220/18/9/2933/s1, Figure S1: Custom-built 7 × 5 well type for the measurements. Upper left: well plate containing reaction solutions, lower left: membrane holders placed in the wells, right: assembling parts for the holder and plate, Figure S2: Custom-made membrane holder (left) and fabricated electrodes on glass wafer (right). Capture antibodies were immobilized on the MD and the MD were stationed between holder and its lid.
