# Supplementary material for: Membrane-Based Microwave-Mediated Electrochemical Immunoassay for the In Vitro, Highly Sensitive Detection of Osteoporosis-Related Biomarkers
Source: Sensors (Basel). 2018 Sep 4;18(9):2933. doi: 10.3390/s18092933 (PMC6163632; doi:10.3390/s18092933)
Supplement: Supplementary file 1 [file sensors-18-02933-s001.pdf]

Membrane-based microwave-mediated electrochemical immunoassay for the in vitro, highly sensitive detection of osteoporosis related biomarkers

Hye Youn Kim [1], Shinobu Sato [2], Shigeori Takenaka [2], and Min-Ho Lee [1,*]

[1] School of Integrative Engineering Chung-Ang University, 84 Heukseok-Ro, Dongjak-Gu, Seoul 06974, Korea;

[2] Department of Applied Chemistry, Research Center for Biomicrosensing Technology, Kyushu Institute of Technology, Fukuoka, 804-8550, Japan;

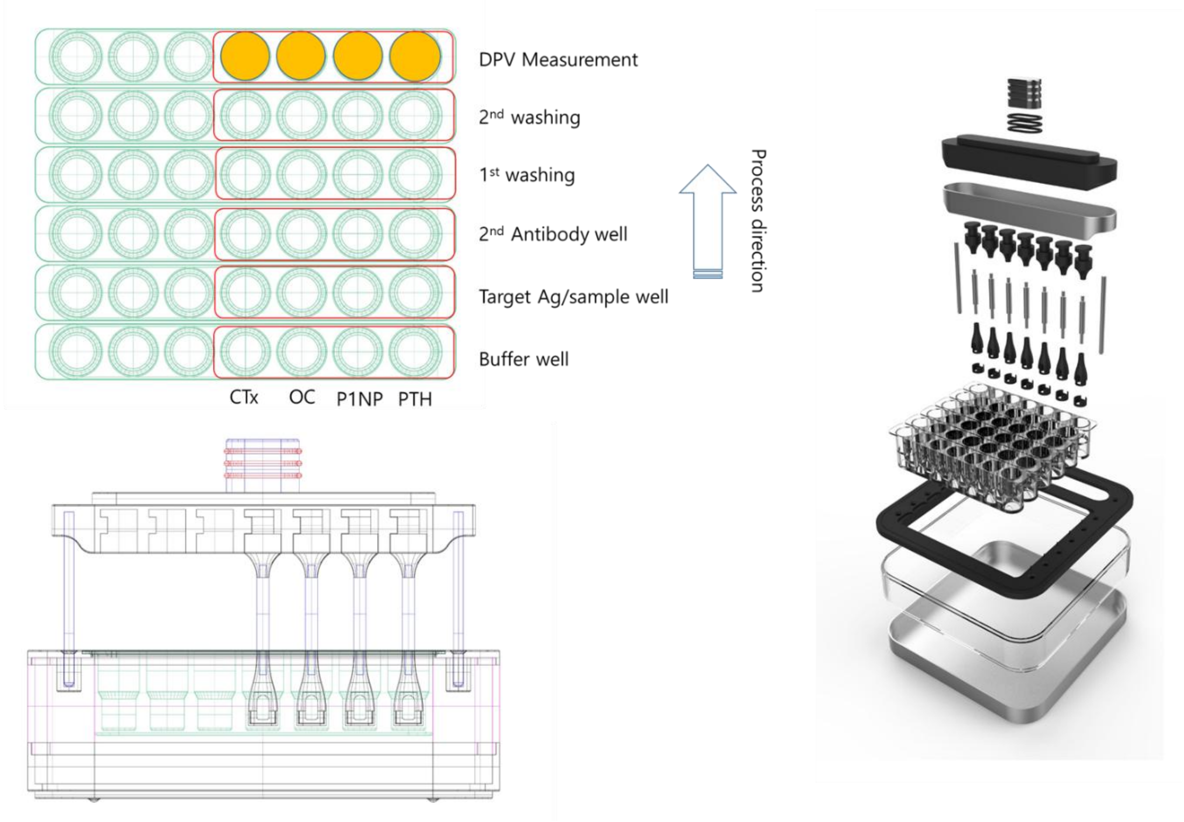

Figure S1. Custom-built 7 x 5 well type for the measurements. Upper left: well plate containing reaction solutions, lower left: membrane holders placed in the wells, right: assembling parts for the holder and plate.

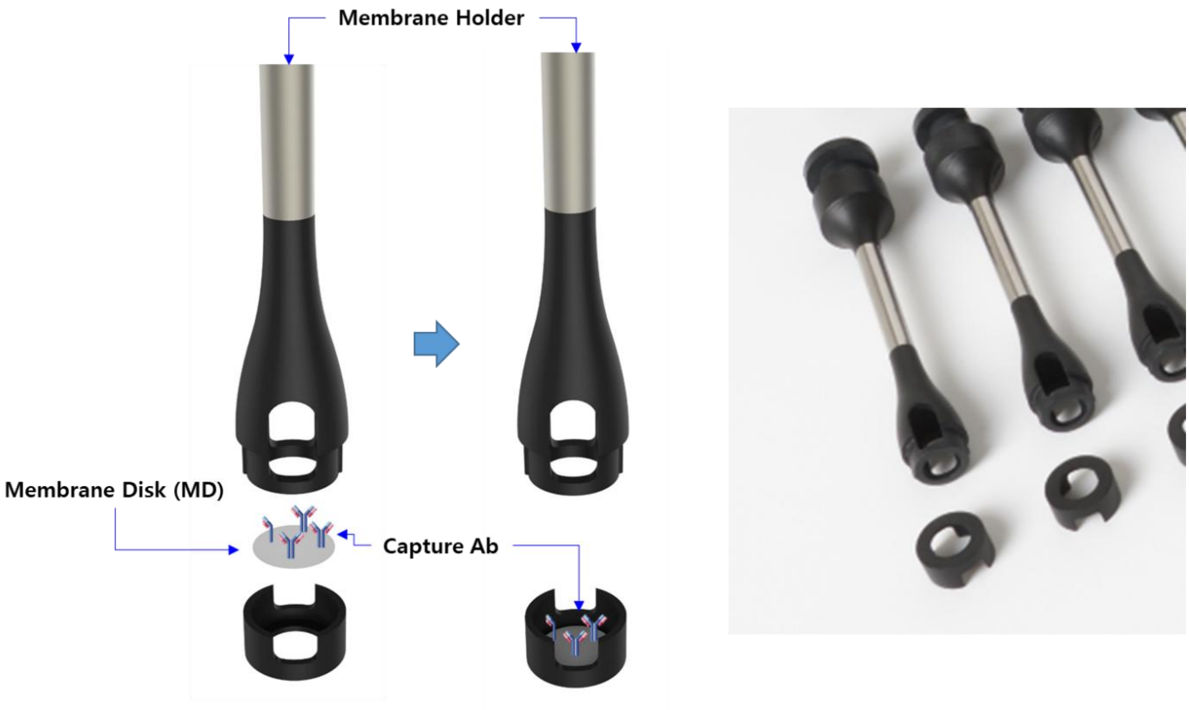

Figure S2. Custom-made membrane disk holder. Capture antibodies were immobilized on the MD and the MD were stationed between holder and its lid.

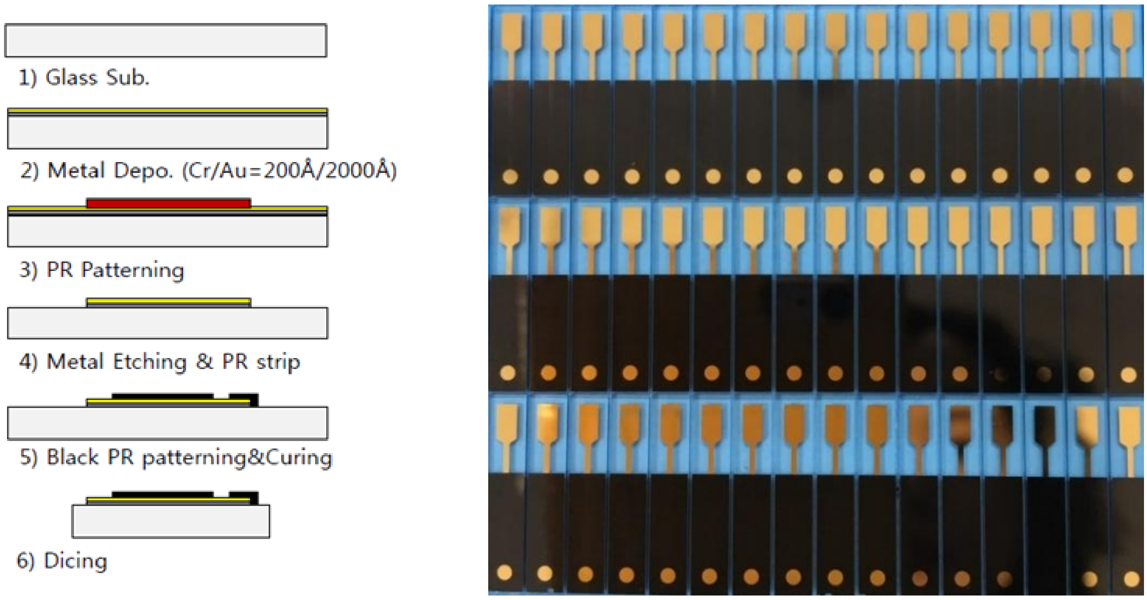

Figure S3. Fabrication process of electrodes (left) and the electrodes on glass wafer (right).
